# Viral social media videos can raise pro-social behaviours when an epidemic arises

**DOI:** 10.1007/s40881-021-00104-w

**Published:** 2021-09-01

**Authors:** Yiting Guo, Jason Shachat, Matthew J. Walker, Lijia Wei

**Affiliations:** 1 Economics and Management School, Wuhan University, Wuhan, 430072 HUB China; 2 Department of Economics and Finance, Durham University Business School, Durham, DH1 3LB UK; 3 Newcastle University Business School, Newcastle Upon Tyne, NE1 4SE UK

**Keywords:** Social media, Pro-sociality, Risk preference, Experiment, Induced emotions, Covid-19, C93, H12, I12

## Abstract

Access to information via social media is one of the biggest differentiators of public health crises today. During the early stages of the Covid-19 outbreak in January 2020, we conducted an experiment in Wuhan, China to assess the impact of viral social media content on pro-social and trust behaviours and preferences towards risk taking with known and unknown probabilities. Prior to the experiment, participants viewed one of two videos that had been widely and anonymously shared on Chinese social media: a central government leader visiting a local hospital and supermarket, or health care volunteers transiting to Wuhan. In a control condition, participants watched a Neutral video, unrelated to the crisis. Viewing one of the leadership or volunteer videos leads to higher levels of pro-sociality and lesser willingness to take risks in an ambiguous situation relative to the control condition. The leadership video, however, induces lower levels of trust. We provide evidence from two post-experiment surveys that the video’s impact on pro-sociality is modulated by influencing the viewer’s affective emotional state.

**Supplementary Information:**

The online version contains supplementary material available at 10.1007/s40881-021-00104-w.

## Introduction

On January 23, 2020, local authorities in China imposed a full lockdown of Wuhan city in response to the emergence of a novel coronavirus and associated disease, Covid-19. This event was followed shortly after by the lockdown of other cities across Hubei province. With the movements of over 50 million people in the centre of China being closely monitored, this represented one of the largest forced quarantines in human history.^1^ The lockdown of Wuhan city was the first, and one of the most stringent, to be implemented globally. During those early days of uncertainty, with scant reporting in official media outlets, social media became a key source of information about the virus for ordinary Chinese citizens. The currency of social media are viral videos and associated messages; during this time, many videos were circulated in private chat groups, showing evocative scenes of containment and relief efforts.^2^

Access to information via social media is one of the biggest differentiators of pandemics today from the past (Balinska & Rizzo, 2009). Social media has become an important medium that individuals turn to for information in public emergencies (Reuter & Kaufhold, 2018). Since information systems, rather than personal experience, are the most likely source of information during a crisis, social media is an important agent of risk amplification (Kasperson & Kasperson, 1996; Pidgeon et al., 2003). Recent research recognises the potential of viral social media content to promote collective action in moments of crisis, alongside possible negative effects for trust and risk perception (Alexander, 2014; Haushofer & Metcalf, 2020; Taylor et al., 2012). During a public health crisis, the coordination of individual efforts towards collective demands underpins the success of mitigating measures (Van Bavel et al., 2020). Trust and risk attitudes are associated with the adoption of health behaviours and so indirectly for controlling the rate of disease transmission (Chuang et al., 2015). It is, therefore, important to understand how social media content influences pro-social, trust and risk-related preferences.

This study examines the influences of social media content on such preferences via the modulation of a viewer’s affective emotional state. A long literature in psychology emphasises that affective states influence normative judgements and decision-making processes (Forgas, 1995; Loewenstein & Lerner, 2003; Pham, 2007).^3^ Online media content that arouses emotions is more likely to go “viral” in the first place (for an example of this in the Chinese social media context, see Fan et al., 2014). Thus, we conjecture that social media videos of the type used in this study may act as an incidental (that is, not normatively relevant for the judgement at hand) influence on individuals’ emotions and mood, which in turn affects their decisions.^4^

An extensive experimental economics literature explores the ability of induced emotions to affect strategic behaviour and individual preferences. Capra (2004) finds that induced good mood leads to more altruistic giving. Drouvelis and Grosskopf (2016) and Bartke et al. (2019) observe that induced positive (negative) affective states increase (diminish) pro-social behaviour in a public goods game, while findings for cooperation are mixed (Hertel & Fiedler, 1994; Hertel et al., 2000). Dunn and Schweitzer (2005) suggest that incidental anger reduces trust. In a gift-exchange game, Kirchsteiger et al. (2006) observe that those individuals who watched a funny movie clip are more generous, while those who watched a sad clip are more reciprocal. Kugler et al. (2012) show that induced mood can have a systematic impact on individual risk preferences: in their study, fearful participants make more risk-averse lottery choices than angry participants.

Our study consists of an economic experiment that measures the behavioural impact of watching viral social media videos, and two follow-up surveys that assess the emotional states induced by the content of those same videos. In the experiment, Wuhan-based students complete a panel of decision tasks to measure the effects of crisis-related social media video stimuli on their pro-social, cooperative and trusting behaviour, as well as their preferences towards risk taking with known and unknown probabilities. The experiment was implemented in late January 2020, the time of greatest uncertainty about the coronavirus in China. All experimental tasks are incentive compatible: all choices have monetary rewards proportional to the good outcomes of the tasks.^5^

The experiment comprises two treatment conditions and a control condition, using a between-subjects design. In the treatment conditions, participants are primed with one of two videos, each of which shows evocative content related to the Covid-19 crisis. The first of these videos shows a senior central government official’s visit to a local hospital and a supermarket (henceforth “Leadership video”). The second of these videos shows health care volunteers from other provinces in transit to Wuhan (henceforth “Volunteer video”). Both videos were circulating widely and anonymously among chat groups on the Chinese social media application WeChat at the time of the experiment. In the control condition, participants watch a neutral product advertisement video, unrelated to the crisis (henceforth “Neutral video”).

We find that priming participants with either the Leadership or Volunteer video results in significantly more pro-social behaviour relative to the Neutral video, as measured by participants’ levels of altruism and expectations of reciprocity in the decision tasks. Participants’ also display lesser willingness to take risks in an ambiguous situation in the treatment conditions, although there is no systematic effect of the viral videos on risk preferences in unambiguous situations. The Leadership video alone induces a significant fall in trust, although we caution on drawing generalized conclusions from this result, because we test only one video which involves just one (prominent) government figure.

In the two follow-up surveys, we find evidence to support the argument that the videos impact behaviour through the manipulation of affective emotional states. In the first survey, we ask respondents to select five events, from a list of fifteen, which acted as psychologically positive motivating factors during the early stages of the Covid-19 crisis. The two most selected events are health care teams volunteering to assist in Hubei province and national leaders countering the epidemic. In the second survey, we evaluate the impact of viewing the videos in our experiment through a set of questionnaires that assess an inventory of emotional measurements. We find that the Leadership and Volunteer videos each induce a significant rise in positive affect relative to the Neutral video, with underlying increases in joviality and self-assuredness. Those individuals who watch the Volunteer video also report greater attentiveness. There is some evidence that viewers of the Leadership and Volunteer videos feel a comparatively greater sense of guilt.

The paper continues in Sect. 2 with a description of the experimental design and procedures. In Sect. 3, we present the results of the economic experiment. In Sect. 4, we describe the two post-experiment surveys we undertook to assess the video-induced changes in emotional states. In Sect. 5, we conclude with a discussion of how to interpret the results and outline some limitations of our study.

## Experimental design

**Fig. 1:**
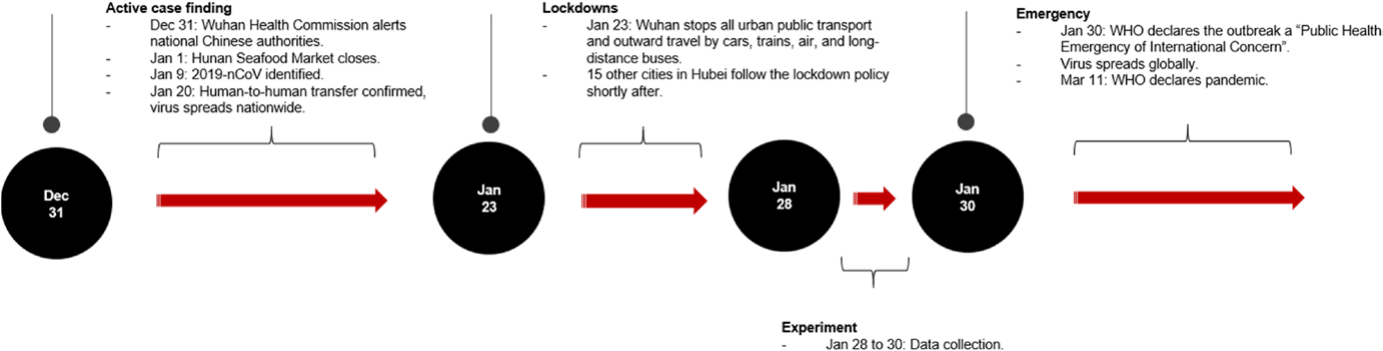
Timeline of Covid-19 events in Hubei province, 2019–2020. Figure shows the dates of experiment data collection in context of wider events relating to the public emergency

A timeline of our economic experiment in context of the Covid-19 outbreak in Hubei province is displayed in Fig. 1. We recruited 240 participants at random from an online database of over 9000 Wuhan University students. Our study has three experimental conditions based upon the nature of a priming social media video. We randomly assigned 80 participants to each condition. The experiment consisted of twelve sessions designed for twenty participants each. Three sessions, one for each experimental condition, were run concurrently in the morning and afternoon on January 28 and 30, 2020. Morning and afternoon sessions differed by which subset of tasks we administered. The final day of data collection coincided with the World Health Organization’s declaration of the virus as a Public Health Emergency of International Concern (WHO, 2020).

Invitations to participate in a session were sent directly to participants’ WeChat accounts, which is the most popular instant messaging app and the largest social media platform in China. At the time of the experiment, participants had already left the university for the semester break, which coincides with the annual spring festival holiday. Thus, participants were residing in twenty-nine different provinces. Forty participants were residing in Hubei province, including seventeen from Wuhan. On average, each session lasted forty-five minutes, and participants earned 63.79 RMB (about 9.5 US dollars), including a participation fee of 10 RMB. Participation in the experiment was completed using a mobile device and we transferred payment to their WeChat account immediately after completion of their experimental session.

Four canonical games, multi-persons decision problems, are considered: a dictator game (DG), an ultimatum game (UG), a trust game (TG), and a prisoner’s dilemma game (PD).^6^ In the DG, players are matched into pairs and assigned to the role of player 1 or player 2. Player 1 is allotted a real sum of money (the stake) and decides how to allocate the stake between the two. Higher amounts offered to player 2 reflect greater pro-sociality on the part of player 1. The UG is the same as the DG, except that player 2 can choose to accept or reject—which results in both receiving zero—the proposed allocation. In the UG, higher amounts offered by player 1 may reflect a combination of pro-sociality and expectations of reciprocity; player 2’s decision reflects actual reciprocity. The stake used in the DG is 5 RMB, and in the UG is 8 RMB. We use different stakes to reduce the likelihood of participants anchoring on their first response when making their second.

In the TG, players are again matched into pairs, player 1 is allotted a stake and decides how much of that stake to transfer to player 2. The amount transferred is tripled, which player 1 is aware of, before reaching player 2. After player 2 receives the multiplied transfer, he or she decides how much of it to return to player 1. In the TG, the amount that player 1 sends is a measure of trust and the amount that player 2 returns reflects trustworthiness. The stake used for this game is 8 RMB.

The PD is a normal-form game in which each player chooses to either Cooperate or Defect. Choosing Defect yields a player a higher payoff than choosing Cooperate against each of the opponent’s possible choices. However, the pair’s total payoff is highest when both choose to Cooperate. The payoff to mutual cooperation was 6 RMB, to mutual defection 3 RMB, to unilateral cooperation 0 RMB and to unilateral defection 9 RMB.

We also include tasks designed to elicit preferences towards risk taking with known and unknown probabilities.^7^ The risk preference elicitation task with known probabilities involves a series of nine pairwise choices between a lottery (option A) and a sure amount of money (option B). The lottery remains fixed across all choices: a 50% chance of receiving 9 RMB, and a 50% chance of receiving 3 RMB. The sure amount increases evenly with each choice from 3 RMB up to 9 RMB. The task to elicit preferences over risk taking in an ambiguous situation is identical except that the lottery is unknown. Participants are informed that if they choose option A, a ball is randomly drawn from an opaque urn. The urn contains both red and blue balls, but the number of each colour is unknown. If the draw is red, they earn 9 RMB. If the draw is blue, they earn 3 RMB. One choice from each risk/ambiguity elicitation task is randomly drawn for payment.

Before completing the decision tasks, participants watch a once repeated video of approximately two minutes in length.^8^ To mitigate the possibility of experimenter demand effects, we did not provide any cover story or description to accompany the videos. In the control condition, participants are shown a product advertisement for the sculpting of a plastic bottle, unrelated to the Covid-19 crisis (the Neutral video). By contrast, the two treatment conditions each feature a video that had been circulating widely and anonymously on Chinese social media around the time of the experiment.

In the Leadership video treatment, participants are shown excerpts from the visit of a senior central government official to Wuhan Jinyintan Hospital—the first hospital designated to treat patients with Covid-19—and to a local supermarket, on January 27, 2020. In this video, the senior central government official uses morale-boosting words and phrases such as “protect”, “save lives” and “empowered”, to which the doctors and customers alike respond enthusiastically. In the Volunteer video treatment, participants are shown a video of health care volunteers from other provinces in transit to Wuhan. The video pans to teams from various hospitals and acknowledges their place of origin and number, in almost regimental style. Exclamations are heard over the public tannoy such as “go Wuhan!” and “we will carry the burden with you”.

We summarize our economic experiment and the data collection process in Table 1. The decision-making tasks were completed sequentially. In the morning sessions we excluded the UG task, and in the afternoon sessions we excluded the TG task. We did this to rule out the possibility of a behavioural spillover effect; specifically, the second-movers conditioning their actions on the first movers’ actions in both the UG and TG games. We only informed participants of their respective task outcomes and earnings after all decision tasks were completed. No individual participated in more than one session and all sessions consisted of 20 participants, except one session in which there wereonly 16 participants due to participant no-shows. To ensure consistency of the interface, four participants are excluded from the sample for using a computer rather than mobile device to complete the experiment.

**Table 1:** Experimental design and session information

Treatment	Jan 28 morning	Jan 28 afternoon	Jan 30 morning	Jan 30 afternoon
Neutral video	20 participants	20 participants^a^	20 participants	20 participants
Volunteer video	16 participants	20 participants	20 participants	20 participants
Leadership video	20 participants^b^	20 participants	20 participants	20 participants
Excluded task	Ultimatum game	Trust game	Ultimatum game	Trust game

^a^ID 5 & 20 used a computer and are excluded from the analysis

^b^ID 12 & 18 used a computer and are excluded from the analysis

## Experimental results

The aggregate outcomes of the lab experiment are presented in Table 2. The Leadership (*L*) and Volunteer (*V*) videos have marginally significant positive effects on pro-sociality in the experiment relative to the Neutral (*N*) video. In the DG, both videos increase the average amount sent by dictators (*p* value = 0.08 and *p* value = 0.07, respectively, *n*_L_ = *n*_V_ = 38 and *n*_N_ = 39). There is also evidence that participants offer higher amounts in the UG. While this increase is not significant at conventional thresholds for the Leadership video versus the Neutral video, it is for the Volunteer video (*p* value = 0.12 and *p* value = 0.03, respectively, n_L_ = 20, *n*_V_ = 20 and *n*_N_ = 19). The corresponding difference between Leadership and Volunteer video conditions, however, is not significant (*p* value = 0.54). UG acceptance rates are lower in the Neutral video condition in response to the reduced offers, although higher variability means that the pairwise differences are not significant (*p* value = 0.15 and *p* value = 0.15, respectively, *n*_L_ = 20, *n*_V_ = 20 and *n*_N_ = 19).

**Table 2:** Key outcome measures in the experimental treatments

Task (response range)	*N*^c^	Definitions: higher value implies	Neutral video	Leadership video	Volunteer video
DG amount sent^a^ (0–5)	39/38/38	$\uparrow$ Pro-sociality	1.37 (1.01)	1.76* (0.99)	1.84* (1.03)
UG offer^a^ (0–8)	19/20/20	$\uparrow$ Pro-sociality or expectations of reciprocity norms	2.58 (1.43)	3.23 (1.24)	3.55** (0.83)
UG acceptance rate^b^ (accept = 1, reject = 0)	19/20/20	$\downarrow$ Actual reciprocity norms	0.79 (0.42)	0.95 (0.22)	0.95 (0.22)
TG amount sent^a^ (0–8)	20/18/18	$\uparrow$ Trust	3.20 (2.28)	2.06* (1.98)	3.11 (2.78)
TG return^a^ (0– 24)	20/20/18	$\uparrow$ Trustworthiness	3.05 (3.78)	2.1 (3.38)	3.21 (4.38)
PD cooperation rate^b^ (cooperate = 1, defect = 0)	78/78/76	$\uparrow$ Cooperation	0.40 (0.49)	0.46 (0.50)	0.45 (0.50)
Risk preference^a,d^ (0–9)	78/77/75	$\uparrow$ Willingness to take risks	4.78 (1.54)	4.75 (1.46)	4.79 (1.48)
Ambiguity preference^a,d^ (0–9)	77/74/75	$\uparrow$ Willingness to take risks under ambiguity	4.53 (1.51)	4.14** (1.44)	3.96** (1.33)

*p < 0.1; **p < 0.05; ***p < 0.01. Mean (SD) values are presented in the table

^a^Two-tailed Wilcoxon rank-sum test

^b^Two-proportions *z* test

^c^The convention is number of observations by Neutral/Leadership/Volunteer video treatment

^d^First row in which the certain option was chosen. We excluded responses from participants exhibiting inconsistent preference, defined as switching between the lottery and the sure amount of money options more than once (no participant switched only once in the “wrong direction”, i.e. from a low certain amount to the lottery). For the Leadership video, this required excluding one response for risk and four for ambiguity; for the Volunteer video, this required excluding one response for risk and one for ambiguity; and for the Neutral video, this required excluding no responses for risk and one for ambiguity

Interestingly, the Leadership video appears to undermine trust. Amounts sent by first movers in the TG are lower in the Leadership video treatment than in the Neutral video control, although this difference is only marginally significant (*p* value = 0.09, *n*_L_ = 18 and *n*_N_ = 20). We find no significant negative effect of the Volunteer video on trust (*p* value = 0.82, *n*_V_ = 18 and *n*_N_ = 20) and no significant difference in the raw TG amount sent between the Leadership and Volunteer video (*p* value = 0.26). Reciprocity, in terms of amounts returned, adjusts proportionally in each experimental condition.^9^ Consistent with earlier TG experiments, a trustor’s decision to transfer money is on average a breakeven strategy (Berg et al., 1995). There are no statistically significant pairwise differences in average PD cooperation rates in the respective treatment conditions relative to the control condition (*p* value = 0.52 and *p* value = 0.64, respectively, *n*_L_ = *n*_N_ = 78 and *n*_V_ = 76).

An ancillary question is how the treatment condition videos influence participants’ preferences towards risk taking with known and unknown probabilities. To examine this question, we use the first row in which the certain option was chosen in the risk and ambiguity preference elicitation tasks. For each task, there were nine rows; risk neutrality corresponds to choosing the certain amount for the first time in row five. While neither the Leadership nor Volunteer video significantly influences risk aversion with known probabilities (*p* value = 0.70 and *p* value = 0.85, respectively, *n*_L_ = 77 *n*_V_ = 75 and *n*_N_ = 78), both videos reduce participants’ willingness to take risks in an ambiguous situation (*p* value = 0.03 and *p* value = 0.02, respectively, *n*_L_ = 74, *n*_V_ = 75 and *n*_N_ = 77). We advise against reading into the absolute levels recorded in the ambiguity preference elicitation task, because our implementation did not permit participants to choose the payoff-relevant colour and so this task may also pick up trust in the experimenter.^10^

That participants’ reactions to the treatment condition videos differ across risk and ambiguity domains is not unusual (see also Cavatorta & Groom, 2020). Whereas risk involves known probabilities, ambiguity describes situations in which probabilities are vague. If the treatment videos heightened feelings of uncertainty about the situation in Wuhan in those early days of the Covid-19 outbreak, individuals may have been prone to act more cautiously when the likelihoods of outcomes associated with their actions are unknown. It does not necessarily follow that they would also prefer to take fewer risks. The probabilities of bad outcomes in an emerging public health crisis are far from well-defined.

Due in part to features of our design (e.g. no role uncertainty, alternation of UG and TG tasks across sessions to preclude second-mover learning), our non-parametric analyses above rest on small sample sizes per condition and so lack statistical power to detect effects. To address this issue, we conduct an individual-level regression analysis of our key outcome measures on indicator variables for the two treatment condition videos, while controlling for other demographic and local factors that might be influencing behaviour. We include as covariates the aggregate number of diagnosed virus cases at provincial level, participant gender, cell phone operating system and screen size.

The results of the regression analysis are presented in Table 3.^11^ We find support for the positive impact of the Leadership and Volunteer videos on first-mover offers in the UG, suggesting that the content of these videos increased expectations of reciprocity norms relative to the Neutral video. We also find some support for our observation that the Volunteer video induces a significant increase in giving by dictators in the DG. While the coefficient estimate on the Leadership video indicator is positive, it is not significant at conventional levels. Since we are primarily interested in the effect of the Leadership and Volunteer videos on “pro-sociality” broadly defined, rather than behaviour in a specific task, we pool standardized first-mover data from the DG and UG and control for task indicators in a random-effects model. There is strong statistical evidence that both treatment condition videos cause an increase in pro-sociality relative to the Neutral video.^12^

**Table 3:** Regression analysis of covariance

	Dependent variable					
	DG sent	UG offer	Pro-sociality^d^	Ambiguity preference	TG sent	TG sent
	OLS	OLS	Panel	OLS	OLS	OLS
	(1)	(2)	(3)	(4)	(5)	(6)
Diagnosed cases^a^	0.031 (0.062)	− 0.125 (0.098)	− 0.013 (0.013)	0.023 (0.054)	− 0.363* (0.209)	− 0.460* (0.272)
Volunteer video^b^	0.414* (0.242)	1.200*** (0.387)	0.149*** (0.053)	− 0.578** (0.225)	− 0.503 (0.813)	− 0.409 (0.869)
Leadership video	0.334 (0.237)	0.857** (0.435)	0.111** (0.052)	− 0.334 (0.239)	− 1.496** (0.716)	− 1.467* (0.799)
Risk preference						− 0.315 (0.337)
Ambiguity preference						0.097 (0.269)
PD cooperation						0.016 (0.680)
Constant	2.522* (1.042)	3.804 (3.736)	0.654 (0.423)	3.185** (1.564)	6.646 (6.461)	7.891 (5.787)
Wald test stat. for diff in treatment videos^c^	0.11	0.79	0.60	1.04	1.59	1.48
Control variables	Yes	Yes	Yes	Yes	Yes	Yes
Random effects	No	No	Yes	No	No	No
Observations	114	59	118	224	55	54
*R*-squared	0.043	0.158	0.098	0.052	0.153	0.165

*p < 0.1; **p < 0.05; ***p < 0.01. Robust standard errors are shown in the parentheses, calculated using the Huber/White sandwich estimator of variance. Control variables include gender, cell phone operating system and screen size. In regression models (1) and (5), one subject is dropped due to a missing value for the screen size variable; in models (4) and (6), two subjects are dropped for this reason. In model (3), random effects are included at the individual-level

^a^Log transformation of the aggregate number of diagnosed virus cases at the provincial level by the midnight of the previous day, based on data from CDC China

^b^The reference video category is the Neutral video

^c^Linear hypothesis test: coefficient of Volunteer video is equal to coefficient of Leadership video

^d^Standardized outcome data on DG amount sent and UG offer; DG task indicator included as control variable

In Table 3, we further examine the impact of the Volunteer and Leadership video on ambiguity preferences. The Volunteer video has a robust negative effect on willingness to take risks in an ambiguous situation which is significant at the 5% level; the coefficient estimate on the Leadership video is negative but not significant at the 10% level.

To estimate the impact of the treatment videos on amounts sent by first movers in the TG, we first conduct a straightforward OLS regression. The Leadership video has a robust negative effect on trust which is significant at the 5% level. The Volunteer video has no significant effect on trust. We then control for subjects’ decisions in the risk and ambiguity tasks, and in the PD game, to isolate the effect of the videos on trust net of pro-sociality, risk and ambiguity aversion.^13^ The negative impact of the Leadership video on trust remains significant at the 10% level. Scholars in the experimental economics literature have long debated whether risk preferences are a component of trusting behaviour (e.g. Ben-Ner & Halldorsson, 2010; Eckel & Wilson, 2004). The decision to take a “social risk” on the trustworthiness of a stranger in the one-shot TG might be viewed as conceptually closer to a preference over ambiguity (Li et al., 2019). Our results are consistent with the interpretation of the Leadership video—but *not* the Volunteer video—undermining the component of trust which is unrelated to preferences over risk, ambiguity and pro-sociality. There is, however, no significant difference between the Leadership and Volunteer video coefficient estimates. We will return to discuss these differential effects further in Sect. 5.

## Two survey studies on the emotional states induced by the social media videos

**Fig. 2:**
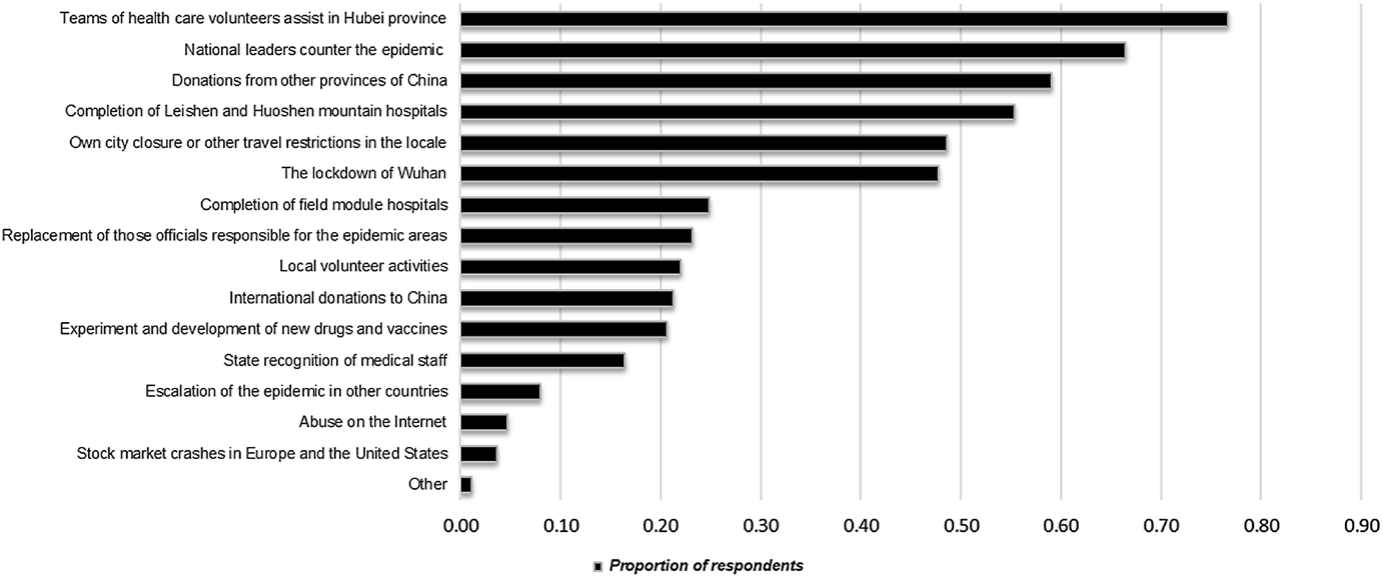
Positive motivating factors during the Covid-19 outbreak. Figure presents the results of a survey question administered from March 12–20, 2020 to 5686 non-student individuals around China. The survey question asked respondents to consider the development of the epidemic and select five factors that had provided them with positive psychological motivation

To gain insight into the channels mediating the differential effects observed in our economic experiment, we provide two post-experiment assessments on how the content of the social media videos are perceived. The first assessment is a follow-up survey we administered from March 12 to 20, 2020 to 5,686 non-student individuals around China. Among other questions, we asked respondents to select five events, from a list of fifteen, which acted as psychologically positive motivating factors in the early stages of the Covid-19 crisis (see Fig. 2).^14^ The two most selected events were health care teams volunteer to assist in Hubei province and national leaders countering the epidemic (77.67% and 66.43%, respectively).

In the second assessment, we evaluate the impact of video viewing on a more extensive inventory of emotional measurements.^15^ We recruited 241 new participants between September 11 and 18, 2020, from the same population of Wuhan-based students as our original experiment. As before, we randomly assigned 80 participants to each experimental condition (81 to the Leadership video condition) and used WeChat for the experimental implementation. At the start of the survey each participant watched their randomly assigned video twice. Participants were told only that the video they were about to watch was recorded and edited within a week of Wuhan’s January lockdown.

After watching their randomly assigned video, participants were asked to complete three psychological questionnaires. These were the Positive and Negative Affect Schedule (PANAS, Watson et al., 1988), Interpersonal Reactivity Index (IRI, Davis, 1983) and Sense of Control (SOC, Lachman & Weaver, 1998).^16^ The PANAS contains two higher-order scales measuring positive and negative affect, which can be decomposed into sub-scales measuring specific positive emotions (e.g. fear, hostility, guilt) and negative emotions (e.g. joviality, self-assurance, attentiveness). The IRI is often used in the social sciences to measure pro-social behaviours along four dimensions: empathy, perspective taking, personal distress and fantasy (e.g. Büchner et al., 2007; Carlo et al., 1999). The SOC provides a generalized measure of competence and contingency along two dimensions: personal mastery and perceived constraints. All responses are measured on Likert scales, with a higher number on the scale indicating greater agreement with the described feeling or emotion at the present moment. The sequence in which the three questionnaires were presented to participants in the experiment was randomized, to control for possible order effects. On completion of the psychological questionnaires, participants were asked to fill in demographic information.^17^ They received a show-up fee of 5 RMB, and a payment of 8 RMB for each questionnaire completed. The total completion time was less than 30 min.

We find significant increases in positive affect induced by the Leadership and Volunteer videos relative to the Neutral video (Table 4). Participants in both treatment conditions describe feeling relatively more self-assured and jovial (all pairwise comparison *p* values < 0.01). There is some evidence that participants who watched the Volunteer video also feel more attentive than those who watched the Neutral video (*p* value = 0.08), and more active, inspired and interested than those who watched the Neutral video (*p* value < 0.01) or Leadership video (*p* value = 0.06).^18^

**Table 4:** Positive and Negative Affect Schedule (PANAS) outcomes

Item	Emotions	Neutral video (*N* = 80)	Leadership video (*N* = 81)	Volunteer video (*N* = 80)
*Higher-order scales*				
Positive affect (10–50)	Active, alert, attentive, determined, enthusiastic, excited, inspired, interested, proud, strong	29.01 (8.48)	32.38*** (6.88)	33.82*** (7.24)
Negative affect (10–50)	Afraid, scared, nervous, jittery, irritable, hostile, guilty, ashamed, upset, distressed	19.69 (7.99)	20.68 (7.61)	20.88 (8.15)
*Positive emotion scales*				
Self-assurance	Proud, strong	5.38 (2.05)	6.58*** (1.87)	7.01*** (1.95)
Attentiveness	Alert, attentive, determined	8.78 (2.67)	9.37 (2.09)	9.55* (2.38)
Joviality	Enthusiastic, excited	5.95 (1.94)	6.69*** (1.73)	6.73*** (1.86)
Other	Active, inspired, interested	8.91 (2.87)	9.74* (2.47)	10.53***^,#^ (2.39)
*Negative emotion scales*				
Fear	Afraid, scared, nervous, jittery	7.99 (3.77)	8.37 (3.74)	8.63 (3.70)
Hostility	Hostile, irritable	3.38 (1.54)	3.14 (1.39)	3.19 (1.50)
Guilt	Guilty, ashamed	3.82 (1.79)	4.28* (1.97)	4.34* (1.77)
Other	Upset, distressed	4.51 (2.19)	4.89 (2.01)	4.73 (2.28)

*^[#]^*p* < 0.1; **p < 0.05; ***p < 0.01; based on two-tailed Wilcoxon rank-sum test versus the Neutral [Leadership] video. Mean (SD) values are presented in the table

By contrast, we find no significant difference in negative affect for either the Leadership video (*p* value = 0.42) or Volunteer video (*p* value = 0.35), and little underlying variability in the negative emotions of fear or hostility. An interesting exception is that participants in both the Leadership and Volunteer video conditions describe feeling a greater sense of guilt than their counterparts in the Neutral video condition (respectively, *p* value = 0.07 and *p* value = 0.08). We observe no systematic differences in responses to the IRI or SOC questionnaires.^19^

The implied positive relationship between positive affect and pro-sociality in the economic experiment is consistent with prior studies (Bartke et al., 2019; Capra, 2004; Drouvelis & Grosskopf, 2016). The relative effect sizes also support this interpretation. Compared to the Neutral video, the Volunteer video induced a larger emotional response across all four positive emotion sub-scales than the Leadership video (see second panel of Table 4), which translated into consistently larger behavioural estimates of the video’s impact on pro-sociality (see Table 2 and columns 1–3 of Table 3).

Neither treatment condition video significantly affected fear, which Kugler et al. (2012) observed to increase risk aversion, and we find no significant difference in risk preferences versus the control condition. While it is plausible that heightened feelings of guilt are behind the fall in willingness to take risks in an ambiguous situation, we do not know of a precedent for this channel and cannot disentangle it from the corresponding increase in positive affect. The elicited emotions are also unable to explain the fall in trust induced by the Leadership video alone, as we discuss below.

## Discussion and conclusion

The results of our economic experiment suggest that viral social media videos, a distinctive feature of modern crises, can influence behaviour and preferences. We find that, in the early days of the Covid-19 outbreak in China, videos showing either a prominent government leader’s visit to Wuhan or the inward transit of health care volunteers induce greater pro-sociality, relative to a neutral video unrelated to the crisis. In the field, this could translate to a rise in donations, assistance and willingness to comply with mandated health behaviours. On the other hand, the Leadership video used in our study had the unintended consequence of decreasing individuals’ levels of trust. This may undermine the authorities’ effectiveness in crisis response efforts.

It is important to keep in mind that these videos are context-specific and not representative of the macrocosm of social media videos circulating on Chinese social media platforms during January 2020. We cannot rule out that participants’ perceptions of the Leadership video are biased by their preconceptions about the particular leader involved. One should remain wary of extrapolating our findings as to the effect of viral media content on behaviour to other populations.

We also uncover evidence that the social media videos used in our study had an incidental influence on individuals’ emotions. Specifically, the Leadership and Volunteer videos induced significant increases in positive affect and mood, which may plausibly have driven the increase in pro-social behaviours. This complements evidence from hypothetical survey response data that emotional responses to a pandemic correlate with intended compliant behaviours (Rubin et al., 2010; Sadique et al., 2007). Two *caveats* should be noted here.

First, our measurements of emotional responses to the video stimuli were elicited nearly 8 months after the behavioural measures obtained in the economic experiment. Thus, it is possible that the videos would have induced different emotional responses between the two time periods. In this regard, we take re-assurance from the consistency of our psychological response data with the representative follow-up survey conducted in early March 2020, which indicate that the events broadcast in the two social media videos were perceived among ordinary Chinese citizens as positive motivating factors.

Second, we observe few systematic differences in emotional responses between the Leadership and Volunteer videos themselves, and so emotions cannot well explain the differential impact of the Leadership video on trust. An alternative explanation for this finding is that the content of the two videos had differential cognitive (rather than affective) influences. Li et al. (2019) show that participants’ inability to discriminate between events with different likelihoods dampens their tendency to act based on beliefs; conditional on equally optimistic beliefs about the other’s trustworthiness, the more insensitive individuals are less inclined to trust. If, as implied by our behavioural measurements, the Leadership and Volunteer videos increased participants’ pro-sociality, but decreased their willingness to take risks under ambiguity, then the net outcome for trust may be a null effect (as observed in the Volunteer video). If the Leadership video further reduced the ability of participants to discriminate between different likelihood levels, then a negative impact on trust would result in this condition.

A further possibility is that the source of the video influences behaviour. The direction of communication in the Leadership video is from leader to citizen, whereas the direction of communication in the Volunteer video is citizen to citizen. Leadership studies have traditionally suggested a top–down communication style is most effective in establishing trust and conformity (the *centralization thesis*, e.g. 't Hart et al., 1993; McEntire & Dawson, 2007). Other literature has challenged the veracity of the *centralization thesis* in times of crisis, postulating that bottom–up communication styles are more effective (Boin and 't Hart, 2003; Demiroz & Kapucu, 2012). On this interpretation, our experimental findings on trust would come out in favour of the critics.

## Supplementary Information

Below is the link to the electronic supplementary material.

Supplementary file1 (PDF 675 KB)

## Data Availability

Data files are available from the authors on request. Experimental instructions and further details are contained in the Supplementary Material.

## Footnotes

1 See, for example, https://apnews.com/7f7336d2ed099936bd59bf8cb7f43756.

2 See, for example, https://www.nytimes.com/2020/01/24/world/asia/china-social-media-coronavirus.html.

3 Various experimental economics studies also find a correlation between emotions and behaviour (Bolle et al., 2014; Charness & Grosskopf, 2001; Hopfensitz & Reuben, 2009; Joffily et al., 2014; Meshulam et al., 2012; Sanfey et al., 2003; Schniter & Sheremeta 2014; Xiao & Houser, 2005).

4 Psychologists distinguish between emotions (intense and short-lived feelings) and mood (diffuse feelings that tend to persist). For a discussion, see Lerner et al. (2015). We do not pursue this distinction further here.

5 This contrasts with psychometric-style questionnaires conducted during previous public health emergencies (Bults et al., 2011; Jones & Salathé, 2009; Leung et al., 2005; Prati et al., 2011; van der Weerd et al., 2011).

6 An additional two games were implemented to elicit measures of cognitive reasoning and perceptions of strategic uncertainty. Since these games are not directly related to pro-sociality, we do not report on them here. Details of these games are available in the Supplementary Material.

7 An additional four tasks were implemented to measure preferences for truth-telling, time preferences and aversion to losses. Details are contained in the Supplementary Material; for a discussion of some related results using a different dataset, see Shachat et al. (2021).

8 Specifically, the Leadership video lasts 2 min and 3 s, the Volunteer video lasts 1 min and 47 s, and the Neutral video lasts 1 min and 54 s. The videos used in this study (subtitled in English) and corresponding transcripts are available from the authors on request.

9 We also exclude observations in which first movers sent zerorns and calculate a measure of trustworthiness based on returns relative to the amount sent; there is no significant pairwise difference in this measure between any two of the three conditions.

10 Since we focus on relative comparisons, this is a relevant confound only if the videos had differential impacts on participants’ trust in the experimenter. While we cannot rule this out, we observe a (marginally significant) fall in generalized trust in the Leadership video condition, but not the Volunteer video condition, and so if this were a confound, we might expect to observe a larger negative effect on risk taking under ambiguity in the Leadership video condition. Yet we observe comparable effects of the two videos.

11 In the Supplementary Material, we also present regression analyses for second-mover behaviour in the TG and UG, respectively (Table S2). Amounts returned/acceptance rates are significantly increasing in the first-mover amount sent/offered, but there is no differential impact by treatment video. In a further set of regressions, we find no significant impact of the Leadership or Volunteer video on risk preferences and PD cooperation rates (Table S3), nor on pooled risk and ambiguity decisions in a random effects model (Table S4).

12 We are grateful to the editors for this suggestion. Random effects are included at the individual-level. Since the regression results reported in column 3 of Table 3 are based only on those subjects who played both the DG and the UG, the change in statistical significance on the Leadership video coefficient may be driven by a change in sample rather than by a treatment effect. To check this possibility, we compare giving in our sample between participants in the morning and afternoon sessions; there is no significant difference in mean DG amount sent between the morning subsample (1.53) and afternoon subsample (1.78) based on a two-tailed Wilcoxon rank-sum test (*p* value = 0.28).

13 In the Supplementary Material, we also present the results from a two-step regression to proxy for aspects of trust not directly captured by the tasks implemented (Table S5). The results are qualitatively unchanged. A limitation of the regression approach to decomposing trust is that measurement error may be a non-trivial confound (Gillen et al., 2018). Further drawbacks are the restrictive assumption of a linear relationship and potential omitted variable bias. An alternative, non-parametric approach is to use exogenous treatment variation (Engelmann et al., 2021).

14 Full details of the survey administration are included in the Supplementary Material.

15 We thank an anonymous reviewer for this suggestion.

16 The complete questionnaires are contained in the Supplementary Material.

17 Demographic characteristics of this follow-up sample are comparable to the main experiment (Tables S1 and S6).

18 We observe no other significant difference in emotional response between the Leadership and Volunteer video treatments.

19 Results of the IRI and SOC can be found in the Supplementary Material (Table S7).

## Code availability

Computing scripts to replicate the data analyses are available from the authors on request.
