# Supplementary material for: Viral social media videos can raise pro-social behaviours when an epidemic arises
Source: J Econ Sci Assoc. 2021 Sep 1;7(2):120–38. doi: 10.1007/s40881-021-00104-w (PMC8409073; doi:10.1007/s40881-021-00104-w)
Supplement: Supplementary file 1 — Supplementary file1 (PDF 675 KB) [file 40881_2021_104_MOESM1_ESM.pdf]

Electronic Supplementary Materials

"Viral social media videos can raise pro-social behaviours when an epidemic arises"

Yiting Guo[a], Jason Shachat[b], Matthew J. Walker[c], and Lijia Wei[d].

[a] Economics and Management School, Wuhan University, Wuhan, HUB 430072, China.
[b] Corresponding author: jason.shachat@durham.ac.uk. Department of Economics and Finance, Durham University Business School, Durham, DH1 3LB, United Kingdom; and Economics and Management School, Wuhan University, Wuhan, HUB 430072, China. https://orcid.org/0000-0002-9719-4555.
[c] Newcastle University Business School, Newcastle Upon Tyne, NE1 4SE, United Kingdom. https://orcid.org/0000-0002-3863-0564.
[d] Economics and Management School, Wuhan University, Wuhan, HUB 430072, China.

This file includes:

Detailed methods.
Experimental instructions and tasks.
Details on experiment videos.
Experiment recruitment message.
Experiment covariates.
Additional regression analyses.
Follow-up assessment 1: survey administration.
Follow-up assessment 1: questions and invitation text.
Follow-up assessment 2: psychological questionnaires.
Follow-up assessment 2: additional results.

Figures:

Fig. S1. Experiment sample distribution.
Fig. S2. Follow-up assessment 1: sample distribution.
Fig. S3. Follow-up assessment 1: sample age distribution.
Fig. S4. Follow-up assessment 1: sample gender distribution.
Fig. S5. Follow-up assessment 1: sample education distribution.
Fig. S6. Follow-up assessment 1: sample income distribution.

Tables:

Table S1. Summary of experiment covariates.
Table S2. Regression analysis of covariance: second-mover behaviour.
Table S3. Regression analysis of covariance: risk preferences and cooperation.
Table S4. Risk-related panel regressions.
Table S5. Two-step regression approach to decomposing trust.
Table S6. Follow-up assessment 2: sample demographic information.
Table S7. Follow-up assessment 2: IRI and SOC outcomes.

Auxiliary media files available from the authors on request:

Video S1. The Leadership video.
Video S2. The Volunteer video.
Video S3. The Neutral video.

Detailed Methods

Experimental design. The recruitment of participants, deployment of the experiment tasks and payment transfers were all executed using the cloud-based Ancademy platform for conducting social science experiments (https://www.ancademy.org/). Ancademy is unique in that it is based on the open interface of WeChat, the Chinese multi-purpose messaging, social media and mobile payment app. WeChat is provided by Tencent Inc. and has 1.15 billion users. The majority of users are in the mainland of China. Typically, each person has only one account because the platform asks for ID card and bank card verification. Each WeChat account is tied to a single mobile phone number and the mobile payment facilities within WeChat are the primary delivery of electronic payments in China (https://www.businessofapps.com/data/wechat-statistics/). We recruited participants using Ancademy and sent them invitations to join an experimental session directly to their WeChat accounts. All participants were instructed to complete their participation through their mobile phones. All participants' earning were quickly transferred to their respective WeChat wallets shortly after the completion of their experimental session.

The Ancademy recruitment database contains over 9,000 students from Wuhan University alone. Invitations to participate in one of the experiment sessions were sent out to a randomly selected subset of this database in two waves. The first recruitment wave invited participation in one of the six sessions on January 28th. The second recruitment wave invited participation in one of the six sessions on January 30th. We invited three times the number of participants required, i.e., in each recruitment wave we invited 360 participants to fill the 120 available spaces. The first 120 participants to sign up in each wave secured a place in one of the experiment sessions.

The experimental protocol was approved by the Academy of Humanities and Social Sciences of Wuhan University. We obtained informed consent from all participants. To program the decision-making tasks, we used the experimental software oTree, which enables interactive experiments to be conducted online (Chen et al., 2016). Participants were not informed about the tasks that they would be asked to complete before registering for an experimental session. Participants were informed that they would receive monetary compensation for their participation. At the conclusion of an experimental session, participants collected their payment via the official Ancademy WeChat account by entering a code provided to them at the beginning of the session.

All participants were students at Wuhan University. At the time of running the experiment, students had already left the university for the spring festival holiday. Participants were thus located in 29 of China's 34 provincial regions (see Figure S1). More than one in seven participants were from Hubei province, which has Wuhan as the provincial capital. This is similar to the student demographic profile of Wuhan University. Participant ages range from 17 to 26, while 40% of participants are male and 60% are female.

Sampling strategy, randomization, and data exclusions. We followed a strategy of random sampling and random assignment to clusters of sessions, and within clusters random assignment to roles in multi-person decision tasks. Due to four participant no-shows, the final sample size is 236. The sample size was chosen to achieve balance across conditions, subject to the constraints of the COVID-19 outbreak environment. Four participants were excluded from the data for using a computer rather than mobile phone to complete the experiment. We excluded these participants because they chose not to follow the experimental instructions,

which explicitly stated to participate through their smartphone. For the risk and ambiguity tasks, we also excluded individuals who submitted inconsistent choices, defined as switching between the lottery and the sure amount of money options more than once. For the Leadership video treatment, this resulted in four exclusions for the risk task and one exclusion for the ambiguity task; for the Volunteer video treatment, this resulted in one exclusion for each of the risk and ambiguity tasks; and for the Neutral video treatment, this resulted in zero exclusions for the risk task and one exclusion for the ambiguity task. No outliers were identified, and no other exclusions were made. The study was not blinded.

Experimental procedures. All sessions followed the same protocol. Upon accepting an invitation to participate in the experiment, participants received an URL that took them to an active instance of the oTree application. Once all participants had entered the session remotely, they watched one of three two-minute videos, twice. The exact video shown depended on the experimental treatment to which a participant was randomly assigned.

The Leadership video shows a senior central government official's visit to a communicable disease hospital (Jinyintan Hospital) and a supermarket (Wushang Supermarket) in Wuhan on January 27, 2020 – the day before the first experimental session. The Volunteer video shows health care volunteers from other provinces in transit to Wuhan (created online at 2020-01-25). The Neutral video shows the sculpting of a plastic bottle (created online at 2017-03-28). All three videos were culled directly from Chinese social media. The Leadership and Volunteer videos were shared anonymously among WeChat group networks during January 2020. The Neutral video was also shared anonymously from Baidu video. We trimmed each source video to ensure that they were of comparable length, two minutes. The videos used in this study (subtitled in English) and corresponding transcripts are available from the authors on request.

After watching their randomly assigned video two times, participants were redirected to a welcome screen describing the experiment guidelines. Participants were informed about the payment protocol and how to claim the payment at the end of an experimental session. Participants were also told that communication was prohibited. In case of some questions arising during completion of the experiment, participants had to enter their mobile phone number before commencing the tasks.

We employed a comprehensive set of twelve behavioural economics tasks commonly used to measure behaviours and preferences. One task, the Ultimatum Game, was excluded from the morning sessions. Another task, the Trust Game, was excluded from the afternoon sessions. Participants thus completed eleven tasks sequentially in a session. Each task was completed only once.

We report on the following six tasks of relevance to our research question.

- *Dictator Game*. Two-person game. Random matching of participants into pairs within the session. Within a pair, participants are assigned to the role of either player 1 or player 2. Roles are asymmetric. Player 1 is allotted 5 RMB and decides how to allocate this sum of money between the two players in the pair. Player 1's allocation is final. Player 2 has no decision to make.

- *Ultimatum Game*. Two-person game. Random matching of participants into pairs within the session. Within a pair, participants are assigned to the role of either player 1 or player

2. Roles are asymmetric and each player decides sequentially. Player 1 is allotted 8 RMB and decides how to allocate this sum of money between the two players in the pair. Player 2 can choose to accept or reject the allocation. In case of rejection, both players receive zero payoff for the task.

- *Trust Game*. Two-person game. Random matching of participants into pairs within the session. Within a pair, participants are assigned to the role of either player 1 or player 2. Roles are asymmetric and each player decides sequentially. Player 1 is allotted 8 RMB and decides how much of this sum of money to transfer to player 2. Any money transferred is multiplied by a factor of three before reaching player 2. Any money not transferred is kept by player 1. Player 2 observes the multiplied transfer and decides how much of it to return to player 1. Any money not returned is kept by player 2.

- *Prisoner's Dilemma Game*. Two-person game. Random matching of participants into pairs within the session. Within a pair, participants are assigned to the role of either player 1 or player 2. Roles are symmetric and each player decides simultaneously. Each player can choose to Cooperate or Defect. The choices are framed neutrally as options C or D. If both players choose Cooperate, both players earn 6 RMB. If both players choose Defect, both players earn 3 RMB. If one player chooses Cooperate and the other player chooses Defect, the cooperating player earns 0 RMB and the defecting player earns 9 RMB.

- *Risk Preference Elicitation*. Individual decision-making task. Participants are presented with a series of nine pairwise choices between a lottery (option A) and a sure amount of money (option B). The lottery remains fixed across all choices: a 50% chance of receiving 9 RMB, and a 50% chance of receiving 3 RMB. The sure amount increases evenly with each choice from 3 RMB up to 9 RMB. After all choices have been made, the system randomly selects one of the nine pairs of options and, depending on the option chosen for this pair, determines the payoff for the task.

- *Ambiguity Preference Elicitation*. Individual decision-making task. Participants are presented with a series of nine pairwise choices between a lottery (option A) and a sure amount of money (option B). If participants choose the lottery, a ball is randomly drawn from an opaque urn. The urn contains both red and blue balls, but the number of each colour is unknown. If the draw is red, they earn 9 RMB. If the draw is blue, they earn 3 RMB. The sure amount increases evenly with each choice from 3 RMB up to 9 RMB. After all choices have been made, the system randomly selects one of the nine pairs of options and, depending on the option chosen in this pair, determines the payoff for the task.

After completion of the tasks, participants answered a short questionnaire eliciting standard demographic information. Finally, each participant viewed a screen containing his or her decision outcomes and payment to be received for each of the tasks. The session then concluded. All sessions lasted approximately forty-five minutes and payments averaged 63.79 RMB (about 9.5 US dollars), including a participation fee of 10 RMB.

The six tasks correspond to the following task numbers in the experimental instructions: Dictator Game (Task 1), Ultimatum Game (Task 11, afternoon sessions), Trust Game (Task 11, morning sessions), Prisoner's Dilemma Game (Task 5), Risk Preference Elicitation (Task 6), Ambiguity Preference Elicitation (Task 7). For completeness, we include the instructions for six additional tasks on which we collected data in the experimental sessions, but which we do not report here.

Experimental instructions and tasks (translated from the original Chinese).

Experiment guidelines

Thank you for participating in this experiment! This experiment includes 11 tasks followed by a questionnaire. Each task is different and you will complete each task once. In each task, we will pay you the amount that corresponds to your decision. Your final earnings are composed of two parts: a participation fee of 10 RMB and a payment for your performance in each task. You can see the results and payment for each task after finishing all tasks.

Please do not communicate with others during the experiment, and do not interrupt the experiment once in progress. Randomly interrupting the experiment will result in the invalidation of the experimental data, causing serious losses to the laboratory, and will also affect your final earnings. During the experiment, once you have made your decision this cannot be changed. Please make your decisions carefully.

If the experiment cannot be completed successfully due to external factors (such as network interruption, other participants interrupting the experiment, etc.), we will pay you the participation fee of 10 RMB.

You will receive your payment after completion of the experiment through the official account "ancademy". You can withdraw it (enter "ancademy"-Assistant-Account-YANZHI-Withdrawal) to your WeChat account. If you have not already followed the official account "ancademy", please follow it as soon as possible. In this way, we can process your payment without delay!

Please fill in your mobile phone number in the box below, so that we can contact you if there is any problem during the experiment.

Next page

Task 1

The introduction of task 1

In this task, all participants will be randomly divided into groups of two. Two participants in each group will be randomly assigned their roles. One is participant P1, and the other is participant P2.

At the beginning of the task, participant P1 has 5 RMB. Participant P1 decides how much money (X) to pass on to participant P2. The remaining money is owned by participant P1.

Payment calculation formula: Participant P1: 5-X; Participant P2: X

Next page

Your choice

You are the participant P1, please decide how much money to pass on to participant P2.

I will pass to participant P2:

¥

Submit

Your choice

You are the participant P2. You don't need to make any decision in this task.

Next page

Task2

The introduction of task 2

In this task, all participants will be randomly divided into groups of four. You and all other team members are required to choose an integer between 0 and 100 (inclusive). Half of the average value of all selected numbers is the target value, and the participant whose integer is closest to the target value wins. If more than one participant selects the target value, they are both winners.

For example, four participants in one group respectively select A, B, C, D. Half of the average of the four numbers is (A+B+C+D)*1/4*1/2, so the participant whose number is closest to this number wins.

If one participant wins, he will gain 8 RMB in this experiment and the other participants will gain 0 RMB; If more than one participant wins, the winners will divide 8 RMB equally.

Next page

Your Choice

Please enter an integer between 0 and 100 (including 0 and 100):

Submit

Task 3

The introduction of task 3

In this task, you need to randomly choose one integer between 0-9 first and add the integer to the last number of your student number. Please keep the ones digit of the sum in mind. For example,

If you choose 6, and the last number of your student number is 1, you will get number 7.

If you choose 4, and the last number of your student number is 9, then the sum of these two is 13. Keep the ones digit of 13, you will get 3.

After the above process is completed, the system will randomly generate a number between 0 and 9 and display it on the screen. You need to tell us whether the number generated by the system is the same as the number you got in the previous process.

If they are the same, you will get the reward of 5 RMB,
If they are different, you will get nothing.

Next page

Your Choice

The random number generated by the system is: 1
Is the random number generated by the system the same as the number you got in advance?

○ Yes

○ No

Submit

Task 4

The introduction of task 4

In this task, all participants are randomly divided into two-person groups, and you need to choose one of two options: option A or option B, where your decision and that of the other participant jointly determine your payment in this task.

The payoff matrix corresponding to your decision and that of the other participant is as follows. In each cell, the first number (in bold) is your payoff, and the second number is the payoff of the other participant. That is,

- If you choose A, and the other participant chooses A, you will gain 3 RMB, the other participant will gain 3 RMB;
- If you choose A, and the other participant chooses B, you will gain 3 RMB, the other participant will gain 0 RMB;
- If you choose B, and the other participant chooses A, you will gain 0 RMB, the other participant will gain 3 RMB;
- If you choose B, and the other participant chooses B, you will gain 8 RMB, the other participant will gain 8 RMB.

		The other participant	
		A	B
You	A	**￥3.00**<br>￥3.00	**￥3.00**<br>￥0.00
	B	**￥0.00**<br>￥3.00	**￥8.00**<br>￥8.00

Your Choice
According to the payoff matrix, your choice is:
○ A
○ B
Submit

Task 5

The introduction of task 5

In this task, all participants are randomly divided into two-person groups, and you need to choose one of two options: option C or option D, where your decision and that of the other participant jointly determine your payment in this task.

The payoff matrix corresponding to your decision and that of the other participant is as follows. In each cell, the first number (in bold) is your payoff, and the second number is the payoff of the other participant. That is,

- If you choose C, and the other participant chooses C, you will gain 6 RMB, the other participant will gain 6 RMB;
- If you choose C, and the other participant chooses D, you will gain 0 RMB, the other participant will gain 9 RMB;
- If you choose D, and the other participant chooses C, you will gain 9 RMB, the other participant will gain 0 RMB;
- If you choose D, and the other participant chooses D, you will gain 3 RMB, the other participant will gain 3 RMB.

		The other participant	
		C	D
You	C	**￥6.00**<br>￥6.00	**￥0.00**<br>￥9.00
	D	**￥9.00**<br>￥0.00	**￥3.00**<br>￥3.00

Your Choice
According to the payoff matrix, your choice is:
○ C
○ D
Submit

Task 6

The introduction of task 6

Hereinafter, you are presented with nine pairs of options listed on the screen, each of which is a lottery, and for each of which you must choose between "option A" and "option B".

- "option A" has a 50-50 chance of receiving 9 RMB and a 50-50 chance of receiving 3 RMB.
- “option B” receives a certain amount of money.

After you have made all your choices, the system will randomly select one of the nine pairs of options, and depending on which option you choose, A or B, the system will randomly determine your reward in this task according to the specified probability.

For example, if the system randomly selects the ith pair of options,

Then if you choose option A in the ith pair of options, you will have a 50-50 chance of receiving 9 RMB and a 50-50 chance of receiving 3 RMB.

If you choose option B in the ith pair of options, you will receive a certain amount of money that is determined by option B.

Next page

Your choice

Option A	Option B
○ a 50-50 chance of receiving 9 RMB; a 50-50 chance of receiving 3 RMB	○ receive ￥3.00 for sure (Fixed income)
○ a 50-50 chance of receiving 9 RMB; a 50-50 chance of receiving 3 RMB	○ receive ￥3.75 for sure (Fixed income)
○ a 50-50 chance of receiving 9 RMB; a 50-50 chance of receiving 3 RMB	○ receive ￥4.50 for sure (Fixed income)
○ a 50-50 chance of receiving 9 RMB; a 50-50 chance of receiving 3 RMB	○ receive ￥5.25 for sure (Fixed income)
○ a 50-50 chance of receiving 9 RMB; a 50-50 chance of receiving 3 RMB	○ receive ￥6.00 for sure (Fixed income)
○ a 50-50 chance of receiving 9 RMB; a 50-50 chance of receiving 3 RMB	○ receive ￥6.75 for sure (Fixed income)
○ a 50-50 chance of receiving 9 RMB; a 50-50 chance of receiving 3 RMB	○ receive ￥7.5 for sure (Fixed income)
○ a 50-50 chance of receiving 9 RMB; a 50-50 chance of receiving 3 RMB	○ receive ￥8.25 for sure (Fixed income)
○ a 50-50 chance of receiving 9 RMB; a 50-50 chance of receiving 3 RMB	○ receive ￥9.00 for sure (Fixed income)
**Submit**	

Task 7

The introduction of task 7

Hereinafter, you are presented with nine pairs of options listed on the screen, each of which is a lottery, and for each of which you must choose between "option A" and "option B".

- "option A" has a chance of receiving 9 RMB and a chance of receiving 3 RMB, but the probabilities of getting 9 RMB and 3 RMB are unknown. (The specific decision process is as follows: there is an opaque box, which contains both red balls and blue balls; the number of the two kinds of balls is unknown; one ball is randomly drawn from the box; if it is a red ball, you will receive 9 RMB; if it is a blue ball, you will receive 3 RMB).
- “option B” receives a certain amount of money.

After you have made all your choices, the system will randomly select one of the nine pairs of options, and depending on which option you choose, A or B, the system will randomly determine your reward in this task.

For example, the system randomly selects the ith pair of options,

If you choose option A in the ith pair of options, you will get 9 RMB or 3 RMB depending on the colour of the ball extracted from the box.

If you choose option B in the ith pair of options, you will a certain amount of money that is determined by option B.

Next page

Your choice

Option A	Option B
○ Your payoff depends on the colour of the ball extracted from the box	○ receive ￥3.00 for sure (Fixed income)
○ Your payoff depends on the colour of the ball extracted from the box	○ receive ￥3.75 for sure (Fixed income)
○ Your payoff depends on the colour of the ball extracted from the box	○ receive ￥4.50 for sure (Fixed income)
○ Your payoff depends on the colour of the ball extracted from the box	○ receive ￥5.25 for sure (Fixed income)
○ Your payoff depends on the colour of the ball extracted from the box	○ receive ￥6.00 for sure (Fixed income)
○ Your payoff depends on the colour of the ball extracted from the box	○ receive ￥6.75 for sure (Fixed income)
○ Your payoff depends on the colour of the ball extracted from the box	○ receive ￥7.50 for sure (Fixed income)
○ Your payoff depends on the colour of the ball extracted from the box	○ receive ￥8.25 for sure (Fixed income)
○ Your payoff depends on the colour of the ball extracted from the box	○ receive ￥9.00 for sure (Fixed income)
Submit	

Task 8

The introduction of task 8

Hereinafter, you are presented with nine pairs of options listed on the screen and for each of which you must choose between "option A" and "option B". Your choice determines whether you have the opportunity to receive a specified cash payment **6 months later** or **7 months later.**

- "option A": you will receive 100 RMB **6 months later** (for example, if today is January 1st, it will be July 1st 6 months later);
- "option B": you will receive payment of an amount specified by this option **7 months later** (for example, if today is January 1st, it will be August 1st 7 months later).

After finishing the experiment, a lottery is administered to determine whether you receive a cash payment for task 8. You have a one-in-ten chance of receiving a cash payment. Specifically, the system will generate you a random integer from 1 to 10, each of which is equally likely. If the random number generated is 10, you are selected to receive a cash payment for task 8.

If you are selected to receive a cash payment, the system will randomly select one of the 9 pairs of options for this task. Your final payment and payment date will be determined according to the option that you chose in the randomly selected pair (option A or B);

If you are not selected to receive a cash payment, your final payment for task 8 is 0.

Next page

Your choice

Option A	Option B
○You will receive ￥100.00 6 months later;	○You will receive ￥100.00 7 months later;
○You will receive ￥100.00 6 months later;	○You will receive ￥103.00 7 months later;
○You will receive ￥100.00 6 months later;	○You will receive ￥106.00 7 months later;
○You will receive ￥100.00 6 months later;	○You will receive ￥109.00 7 months later;
○You will receive ￥100.00 6 months later;	○You will receive ￥112.00 7 months later;
○You will receive ￥100.00 6 months later;	○You will receive ￥115.00 7 months later;
○You will receive ￥100.00 6 months later;	○You will receive ￥118.00 7 months later;
○You will receive ￥100.00 6 months later;	○You will receive ￥121.00 7 months later;
○You will receive ￥100.00 6 months later;	○You will receive ￥124.00 7 months later;

Submit

Task 9

The introduction of task 9

Hereinafter, you are presented with nine pairs of options listed on the screen and for each of which you must choose between "option A" and "option B". Your choice determines whether you have the opportunity to receive a specified cash payment **today** or **one month later**.

- "option A": you will receive 100 RMB **today**;
- "option B": you will receive payment of an amount specified by this option **one month later** (for example, if today is January 1st, it will be February 1st one month later).

After finishing the experiment, a lottery is administered to determine whether you receive a cash payment for task 9. You have a one-in-ten chance of receiving a cash payment. Specifically, the system will generate you a random integer from 1 to 10, each of which is equally likely. If the random number generated is 10, you are selected to receive a cash payment for task 9.

If you are selected to receive a cash payment, the system will randomly select one of the 9 pairs of options for this task. Your final payment and payment date will be determined according to the option that you chose in the randomly selected pair (option A or B);

If you are not selected to receive a cash payment, your final payment for task 9 is 0.

Next page

Your choice

Option A	Option B
○You will receive ￥100.00 today;	○You will receive ￥100.00 one month later;
○You will receive ￥100.00 today;	○You will receive ￥103.00 one month later;
○You will receive ￥100.00 today;	○You will receive ￥106.00 one month later;
○You will receive ￥100.00 today;	○You will receive ￥109.00 one month later;
○You will receive ￥100.00 today;	○You will receive ￥112.00 one month later;
○You will receive ￥100.00 today;	○You will receive ￥115.00 one month later;
○You will receive ￥100.00 today r;	○You will receive ￥118.00 one month later;
○You will receive ￥100.00 today;	○You will receive ￥121.00 one month later;

○You will receive ￥100.00 today;	○You will receive ￥124.00 one month later;
Submit	

Task 10

The introduction of task 10

Hereinafter, you are presented with nine pairs of options listed on the screen, each of which is a lottery, and for each of which you must choose between "option A" and "option B".

- "option A" has a 50-50 chance of **losing** 9 RMB (that is, deducting the corresponding amount of money from your participation fee) and a 50-50 chance of **losing** 3 RMB.
- "option B" **loses** a certain amount of money.

After you have made all your choices, the system will randomly select one of the nine pairs of options, and depending on which option you choose, A or B, the system will randomly determine your loss in this task according to the specified probability.

For example, if the system randomly selects the ith pair of options,

Then if you choose option A in the ith pair of options, you will have a 50-50 chance of losing 9 RMB and a 50-50 chance of losing 3 RMB.

If you choose option B in the ith pair of options, you will lose a certain amount of money that is determined by option B.

Next page

Your choice

Option A	Option B
○ a 50-50 chance of losing 3 RMB; a 50-50 chance of losing 9 RMB	○ lose ￥9.00 for sure (Fixed loss)
○ a 50-50 chance of losing 3 RMB; a 50-50 chance of losing 9 RMB	○ lose ￥8.25 for sure (Fixed loss)
○ a 50-50 chance of losing 3 RMB; a 50-50 chance of losing 9 RMB	○ lose ￥7.50 for sure (Fixed loss)
○ a 50-50 chance of losing 3 RMB; a 50-50 chance of losing 9 RMB	○ lose ￥6.75 for sure (Fixed loss)
○ a 50-50 chance of losing 3 RMB; a 50-50 chance of losing 9 RMB	○ lose ￥6.00 for sure (Fixed loss)
○ a 50-50 chance of losing 3 RMB; a 50-50 chance of losing 9 RMB	○ lose ￥5.25 for sure (Fixed loss)
○ a 50-50 chance of losing 3 RMB; a 50-50 chance of losing 9 RMB	○ lose ￥4.50 for sure (Fixed loss)

○ a 50-50 chance of losing 3 RMB; a 50-50 chance of losing 9 RMB	○ lose ￥3.75 for sure (Fixed loss)
○ a 50-50 chance of losing 3 RMB; a 50-50 chance of losing 9 RMB	○ lose ￥3.00 for sure (Fixed loss)
Enter	

Task 11 (morning sessions)

The introduction of task 11

In this task, all participants will be randomly divided into groups of two people. One is participant P1, and the other one is participant P2.

At the beginning of the task, participant P1 has an endowment of 8 RMB. Participant P1 decides how much money (X) to pass to participant P2. The amount of money passed on triples before it is handed over to participant P2. After participant P2 receives three times as much money, he decides how much money (Y) to pass back to participant P1.

Payment calculation formula: Participant P1: 8-X+Y; Participant P2: 3X-Y

Next page

Your choice

Your role for this task is participant P1. Now you have an endowment of 8 RMB, please decide how much money you are willing to pass to participant P2.

Please enter a number between 0 and 8:

￥

Submit

Your choice

Your role for this task is participant P2. Participant P1 passed on ￥4.00 to you, so you actually receive￥12.00. Therefore, you now have￥12.00, how much money are you willing to pass back to participant P1?

Please enter a number between 0 and￥12.00:

￥

Submit

Task 11 (afternoon sessions)

The introduction of task 11

In this task, all participants will be randomly divided into groups of two people. One is participant P1, and the other one is participant P2.

At the beginning of the task, participant P1 has an endowment of 8 RMB. Participant P1 decides how much money (X) to pass to participant P2. Participant P2 can accept or reject the proposal.

If participant P2 chooses to accept, the two participants in this group will receive the corresponding amounts of money according to the allocation of participant P1.

If participant P2 chooses to reject, the two participants in this group both receive 0 RMB.

Payment calculation formula:
When participant P2 accepts, participant P1: 8-X; participant P2: X;

When participant P2 rejects, participant P1: 0; participant P2: 0;

Next page

Your choice

You are participant P1. How much money are you willing to pass to participant P2?

[¥]

Submit

Your choice

You are participant P2. Participant P1 decided to pass on ¥3.00 to you. Please choose whether to accept or reject the allocation proposed by participant P1?

- ○ Accept
- ○ Reject

Submit

Questionnaire

Please fill in the following questions truthfully. Examples of specific ways to fill in a few questions about mobile phones: If your phone is Huawei nova5 and you purchased it on July 1st, 2019, you need to fill in "Huawei" in the first question, "Nova5" in the second question, "2019/7" in the third question.

How old are you, please?

[ ]

What is your gender, please?

- ○ male
- ○ female
- ○ other

What is your monthly allowance, please?

- ○ Less than 800 RMB
- ○ 800-1500 RMB
- ○ 1500-2500 RMB
- ○ 2500-4000 RMB
- ○ More than 4000 RMB

What is the annual income of your family, please?

- ○ Less than 30000 RMB
- ○ 30000-100000 RMB
- ○ 100000-200000 RMB
- ○ 200000-400000 RMB
- ○ More than 400000 RMB

Which category of the following includes your major, please?

- ○ Philosophy
- ○ Economics
- ○ Law
- ○ Pedagogy
- ○ Literature
- ○ History
- ○ Natural Science
- ○ Engineering
- ○ Agronomy
- ○ Medicine
- ○ Management
- ○ Art
- ○ Others

What is your mobile phone brand?

What is your mobile phone type?

When did you buy this mobile phone (month/year) ?

Where are you, now?

- ○ Home
- ○ Shopping mall
- ○ Classroom
- ○ Library
- ○ Dormitory
- ○ Others

Which equipment do you use to participate in the experiment?

- ○ Desktop
- ○ Laptop
- ○ PAD
- ○ Smartphone
- ○ Others

Which city are you in, now?

[ ]

Next page

Example results screens

Result of task 1

Your role in task 1 is: **participant P1**.
You have an endowment of **￥5.00**.
You decided to pass on **￥1.00** to participant P2.
So your payment in task 1 is: **￥4.00**

Next page

Result of task 1

Your role in task 1 is: **participant P2**
Participant P1 decided to pass on **￥1.00** to you.
So your payment in task 1 is: **￥1.00**

Next page

Result of task 2

Half of the average of the four numbers is: **2.38**; the number that is closest to this number is: **2**.
The number you chose is: **2**.
Therefore, you are the only winner! Your payment in task 2 is: **￥8.00**.

Next page

Result of task 3

In task 3, your decision is: **The number generated by the system is the same as the number you computed**.
Therefore, your payment in task 3 is: **￥5.00**.

Next page

Result of task 4

Your decision in task 4 is: **B**.
The other participant's decision is: **B**.
Therefore, your payment in task 4 is: **￥8.00.**

Next page

Result of task 5

Your decision in task 5 is: **D.**
The other participant's decision is: **D**.
Therefore, your payment in task 5 is: ￥**3.00**.
Next page

Result of task 6

The system randomly selected the **1st** pair of options in task 6.
You have selected option **B** in this pair of options.
In this decision, the system generates your payment based on the corresponding probability.
Your payment in task 6 is: ￥**3.00**.
Next page

Result of task 7

The system randomly selected the **8th** pair of options in task 7.
You have selected option **A** in this pair of options.
In this decision, the system generates your payment based on the corresponding probability.
Your **payment** in task 7 is: ￥**9.00**.
Next page

Result of task 8

In task 8, the system randomly selected the number **9** for you.
This number isn't 10.
Therefore, you will not receive payment in this task, that is, your **payment** in this task will be ￥**0.00**.
Next page

Result of task 9

In task 9, the system randomly selected the number **2** for you.
This number isn't 10.
Therefore, you will not receive payment in this task, that is, your **payment** in this task will be ￥**0.00**.
Next page

Result of task 10

The system randomly selected the **8th** pair of options in task 10.
You have selected option **B** in this pair of options.
In this decision, the system generates your loss based on the corresponding probability.
Your **loss** in task 10 is: ￥**3.75**.
Next page

Result of task 11 (Trust Game sessions)

Your role in task 11 is: **participant P1**.
Your endowment is: ￥**8.00**.

You chose to pass on **￥3.00** to participant P2.
Participant P2 chose to pass on **￥3.00** to you.
Therefore, your payment in task 11 is:
￥8.00-￥3.00+￥3.00=**￥8.00**
Next page

Result of task 11 (Ultimatum Game sessions)

Your role in task 11 is: participant P1.
You and the participant P2 have a total of ￥8.00.
You decided to pass on **￥3.00** to participant P2.
Your proposal is accepted!
Therefore, your payment in task 11 is: **￥5.00**
Next page

The final earnings of the experiment

The participation fee for this experiment is 10 RMB.

The payment you receive from your decisions during the experiment is: **￥44.25** today.

Therefore, your total payment for the entire experiment, including participation fee, is: **￥54.25** today.

Thank you for your participation. We will transfer you the payment after the end of the experiment through official account “ancademy”. You can withdraw it (enter "ancademy"-Assistant-Account-YANZHI-Withdrawal) to your WeChat account. If you haven’t followed official account “ancademy”, please follow it as soon as possible. In this way, you can receive the payment without delay!
Next page

The experiment ends

The experiment is finished, we will pay the experiment remuneration as soon as possible, please make sure to check this!
Next page

Details on experiment videos

The videos used in this study (subtitled in English) and corresponding transcripts are available from the authors on request.

Video S1. The Leadership video. A senior central government official visits a communicable disease hospital (Jinyintan Hospital) and a supermarket (Wushang Supermarket) in Wuhan on January 27, 2020.

Video S2. The Volunteer video. Health care volunteers from other provinces in transit to Wuhan (created online at 2020-01-25).

Video S3. The Neutral video. The sculpting of a plastic bottle (created online at 2017-03-28).

Experiment recruitment message

Students in Wuhan University are welcome to participate in an experiment!

[Participation requirement] Students of all majors and grades in Wuhan University are welcome. Participants are asked to complete the experiment **only using their mobile phones**.

[Payment] Students who successfully register and actually participate in the experiment will receive a participation fee of 10 Yuan, and they will also get extra payment according to the decisions in the experiment.

[Place] Online experiment, you can participate with your mobile phone!

[Session] Each session will last about 0.5-1 hour. The specific time is as follows:

For a leave of absence, please contact the lab R.A at least 12 hours before the experiment.

Center for behavioral and Experiment Research, Wuhan University
Tel: 15071357336

Experiment covariates

Table S1. Summary of experiment covariates. Data collected from experiment participants, which are included as covariates in the regression analysis of covariance.

Statistic	N	Mean	St. Dev.	Min	Max
Age	232	20.621	1.829	17	26
Proportion male	232	0.397	0.49	0	1
Phone Size (inches)	230	5.936	0.491	4.7	6.67
iOS	232	0.237	0.426	0	1
Diagnosed cases[a]	232	539.935	1,054.15	6	3,554

[a] Number of diagnosed virus cases at the provincial level by the midnight of the previous day, based on data from CDC China.

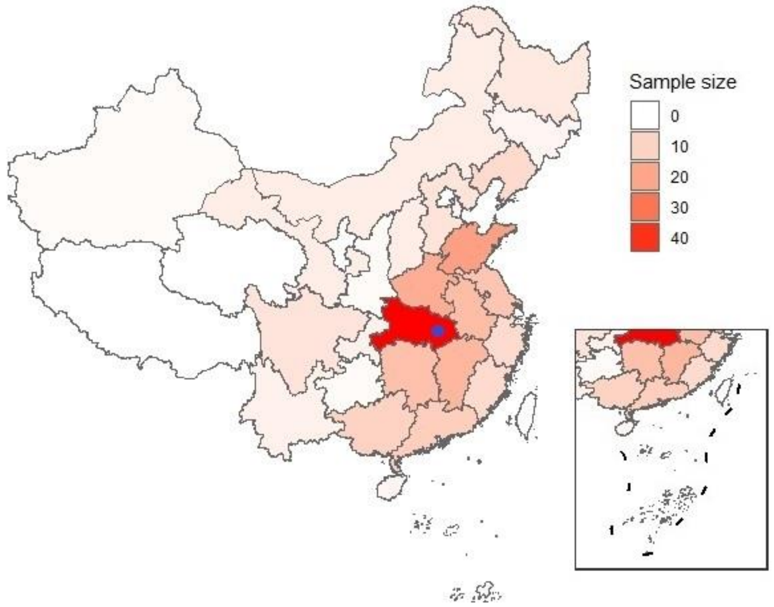

Fig. S1. Experiment sample distribution. The blue dot is Wuhan.

Additional regression analyses

Table S2. Regression analysis of covariance: second-mover behaviour.

	Dependent Variable	
	TG return rate	UG acceptance rate
	OLS	LPM[b]
	(1)	(2)
First-mover send/offer	0.152**	0.096**
	(0.057)	(0.036)
Diagnosed cases[a]	-0.015	0.013
	(0.075)	(0.02)
First-mover send/offer * Volunteer video	0.005	0.001
	(0.058)	(0.028)
First-mover send/offer * Leadership video	-0.015	0.026
	(0.067)	(0.028)
Constant	0.541	0.059
	(2.007)	(0.662)
Control variables	Yes	Yes
Observations	44[c]	59
R-squared	0.24	0.337

Notes: *p<0.1; **p<0.05; ***p<0.01. Robust standard errors are shown in the parentheses, calculated using the Huber/White sandwich estimator of variance. Control variables include gender, cell phone operating system and screen size.

[a] Log transformation of the aggregate number of diagnosed virus cases at the provincial level by the midnight of the previous day, based on data from CDC China.

[b] Linear probability model.

[c] Excludes observations in which the first mover sent zero.

Table S3. Regression analysis of covariance: risk preferences and cooperation.

	Dependent Variable	
	Risk preference	PD cooperation rate
	OLS	Logit
	(1)	(2)
Diagnosed cases[a]	-0.049	0.100
	(0.058)	(0.091)
Volunteer video[b]	-0.005	0.348
	(0.225)	(0.337)
Leadership video	0.014	0.332
	(0.242)	(0.341)
Constant	4.180***	-2.951
	(1.595)	(2.604)
Control variables	Yes	Yes
Observations	228	230
R-squared	0.040	
Log-likelihood		-152.126

Notes: *p<0.1; **p<0.05; ***p<0.01. Robust standard errors are shown in the parentheses, calculated using the Huber/White sandwich estimator of variance. Control variables include gender, cell phone operating system and screen size.

[a] Log transformation of the aggregate number of diagnosed virus cases at the provincial level by the midnight of the previous day, based on data from CDC China.

[b] The reference video category is the Neutral video.

Table S4. Risk-related panel regressions.

	Dependent Variable<br>Risk-related[d]	
	Panel<br>(1)	Panel<br>(2)
Diagnosed cases[a]	-0.001	-0.026*
	(0.005)	(0.015)
Volunteer video[b]	-0.027	-0.035
	(0.020)	(0.042)
Leadership video	-0.013	-0.103**
	(0.022)	(0.041)
Constant	0.350**	0.612*
	(0.142)	(0.325)
Wald test stat. for diff in treatment videos[c]	0.39	2.72*
Control variables	Yes	Yes
Random effects	Yes	Yes
Observations	452	162
R-squared	0.105	0.12

Notes: *p<0.1; **p<0.05; ***p<0.01. Robust standard errors are shown in the parentheses, calculated using the Huber/White sandwich estimator of variance. Control variables include gender, cell phone operating system and screen size. Random effects are included at the individual-level.

[a] Log transformation of the aggregate number of diagnosed virus cases at the provincial level by the midnight of the previous day, based on data from CDC China.

[b] The reference video category is the Neutral video.

[c] Linear hypothesis test: coefficient of Volunteer video is equal to coefficient of Leadership video.

[d] Standardized outcome data on: (1) Pooled decisions in the risk preference and ambiguity preference elicitation tasks; risk preference task indicator included as control variable. (2) Pooled amounts sent in the trust game, decisions in the risk preference and ambiguity preference elicitation tasks; risk and ambiguity preference task indicators included as control variables.

Table S5. Two-step regression approach to decomposing trust.

	Dependent Variable			
	TG sent	Residuals from (1)	TG sent	Residuals from (3)
	OLS	OLS	OLS	OLS
	(1)	(2)	(3)	(4)
Diagnosed cases[a]	-0.476*		-0.458	
	(0.269)		(0.282)	
Volunteer video[b]		-0.254		-0.279
		(0.809)		(0.810)
Leadership video		-1.250**		-1.174*
		(0.605)		(0.611)
Risk preference	-0.345		-0.372	
	(0.298)		(0.309)	
Ambiguity preference	0.225		0.239	
	(0.252)		(0.256)	
PD cooperation			-0.326	
			(0.702)	
Constant	5.242	0.497	4.860	0.479
	(5.030)	(0.455)	(5.324)	(0.462)
Wald test stat. for diff in treatment videos[c]	-	1.73	-	1.39
Control variables	Yes	No	Yes	No
Observations	54	54	54	54
R-squared	0.105	0.059	0.109	0.051

Notes: *p<0.1; **p<0.05; ***p<0.01. Robust standard errors are shown in the parentheses, calculated using the Huber/White sandwich estimator of variance. Control variables include gender, cell phone operating system and screen size.

[a] Log transformation of the aggregate number of diagnosed virus cases at the provincial level by the midnight of the previous day, based on data from CDC China.

[b] The reference video category is the Neutral video.

[c] Linear hypothesis test: coefficient of Volunteer video is equal to coefficient of Leadership video.

Follow-up assessment 1: survey administration

In addition to the experiment, we separately administered an online survey from March 12 to 20, 2020 to non-student individuals around China. Each survey respondent received payment of 2 RMB for their time. We followed a “snowball sampling” strategy. Specifically, we shared the survey in multiple private WeChat groups and asked existing survey participants to re-share the survey among their own colleagues and acquaintances. To ensure that no individual could respond to the survey more than once, we screened responses based on their IP address and WeChat account number.

The survey includes 66 questions in total. Certain sections of the survey contain jump logic and so respondents only needed answer a subset of survey questions. We excluded from the sample any respondents who took less than four minutes to complete the survey. This left a total of 5,686 responses. Survey respondents were based in 32 of China’s 34 provincial regions (Fig. S2). More than one in seven respondents were from Hubei province. The demographic profile of the survey respondents according to age, gender, education and income can be found in Figs. S3 through S6.

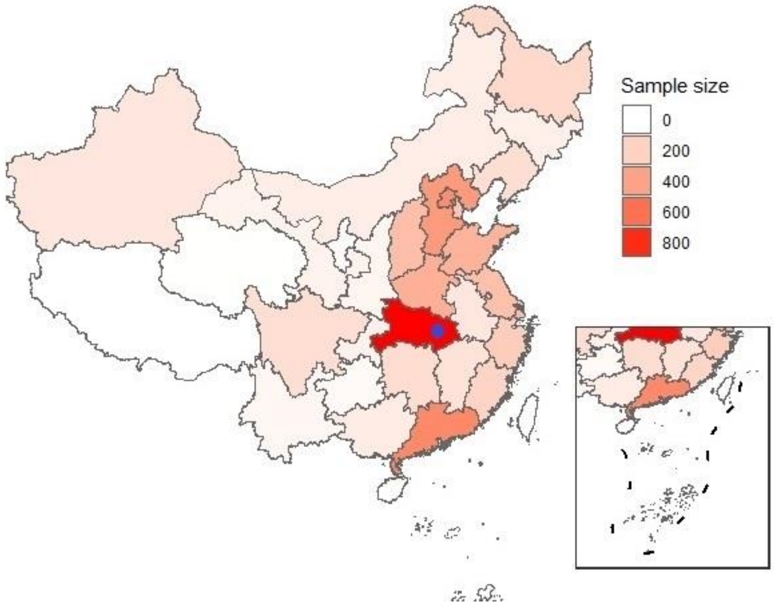

Fig. S2. Follow-up assessment 1: sample distribution. The blue dot is Wuhan.

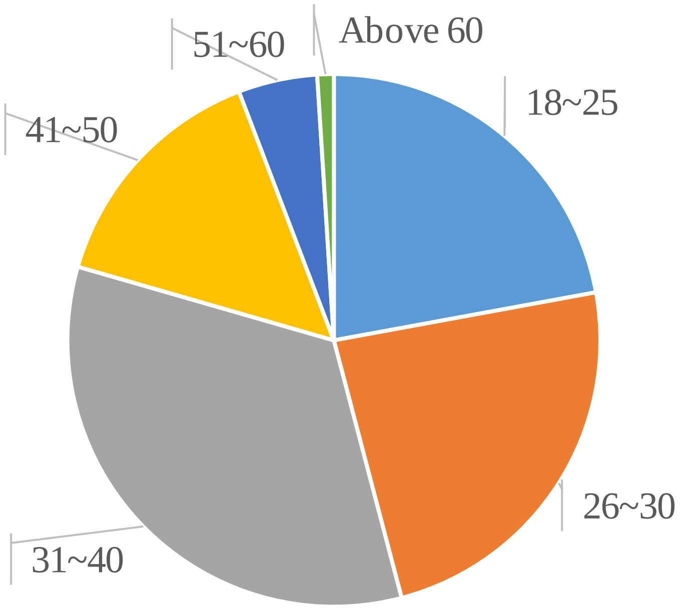

Fig. S3. Follow-up assessment 1: sample age distribution.

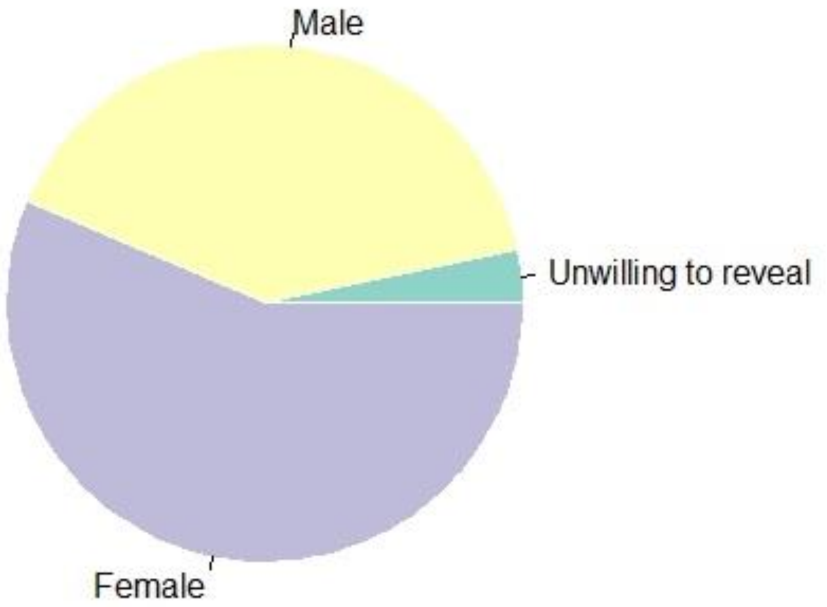

Fig. S4. Follow-up assessment 1: sample gender distribution.

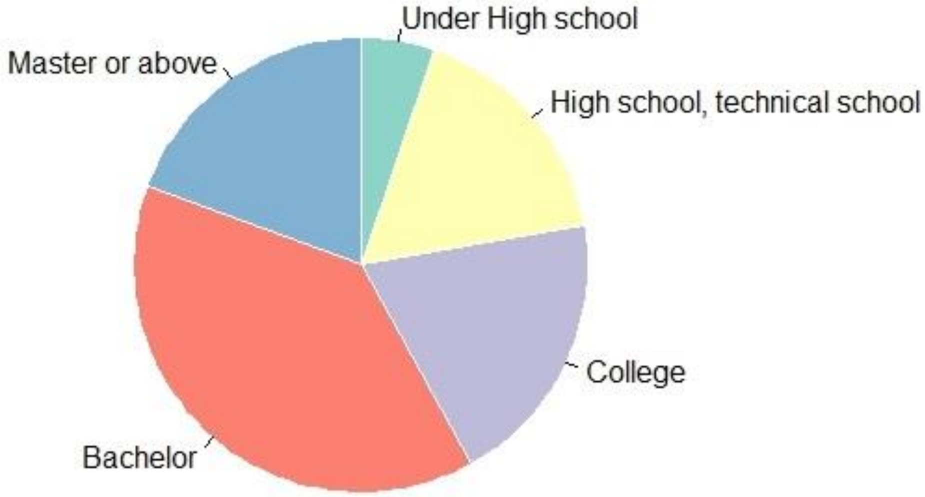

Fig. S5. Follow-up assessment 1: sample education distribution.

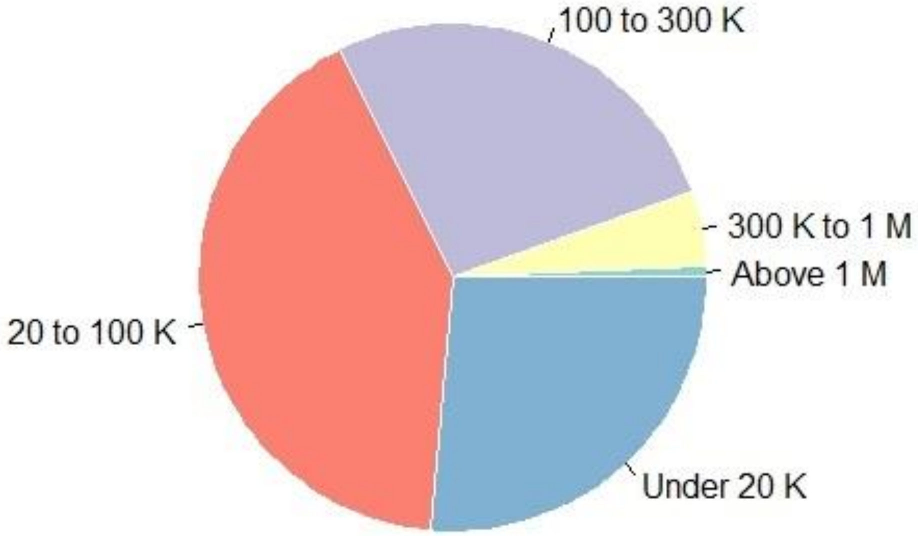

Fig. S6. Follow-up assessment 1: sample income distribution. Units in RMB.

Follow-up assessment 1: questions and invitation text

The full survey questionnaire and invitation text are available from the authors on request.

Follow-up assessment 2: Psychological questionnaires (translated from the original Chinese)

PANAS Questionnaire

In this part, indicate to what extent you feel this way right now. This scale consists of five options that describe different feelings and emotions. For example, there are five numbers 1, 2, 3, 4, 5 to describe "happy". 1 means very slightly or not at all; 2 means a little; 3 means moderately; 4 means quite a bit; 5 means extremely. If you are a little happy, then choose number 2. If you are not happy at all, then choose number 1. It's the same for the other items. There is no right or wrong answer. Be sure to give your answer based on how you feel in the present moment.

	1<br>Very Slightly or Not at All	2<br>A little	3<br>Moderately	4<br>Quite a Bit	5<br>Extremely
1. Interested	1	2	3	4	5
2. Distressed	1	2	3	4	5
3. Excited	1	2	3	4	5
4. Upset	1	2	3	4	5
5. Strong	1	2	3	4	5
6. Guilty	1	2	3	4	5
7. Scared	1	2	3	4	5
8. Hostile	1	2	3	4	5
9. Enthusiastic	1	2	3	4	5
10. Proud	1	2	3	4	5
11. Irritable	1	2	3	4	5
12. Alert	1	2	3	4	5
13. Ashamed	1	2	3	4	5
14. Inspired	1	2	3	4	5
15. Nervous	1	2	3	4	5
16. Determined	1	2	3	4	5
17. Attentive	1	2	3	4	5
18. Jittery	1	2	3	4	5
19. Active	1	2	3	4	5
20. Afraid	1	2	3	4	5

IRI_C Questionnaire

There are 22 items below. How well does each item fit you or how well does each item describe you? 0 = does not describe me at all, 1 = does not describe me well , 2 = neither does nor does not describe me, 3 = describes me well, 4 = describes me exactly. For each item, indicate how well it describes you by choosing the appropriate number from 0~4.

	Does not describe me at all	Does not describe me well	Neither does nor does not describe me	Describes me well	Describes me exactly
1. I often have tender, concerned feelings for people less fortunate than me.	0	1	2	3	4
2. Sometimes I don't feel sorry for other people when they are having problems.	0	1	2	3	4
3. I really get involved with the feelings of the characters in a novel.	0	1	2	3	4
4. In emergency situations, I feel apprehensive and ill-at-ease.	0	1	2	3	4
5. I am usually objective when I watch a movie or play, and I don't often get completely caught up in it.	0	1	2	3	4
6. I try to look at everybody's side of a disagreement before I make a decision.	0	1	2	3	4
7. When I see someone being taken advantage of, I feel kind of protective toward them.	0	1	2	3	4
8. I sometimes feel helpless when I am in the middle of a very emotional situation.	0	1	2	3	4
9. I sometimes try to understand my friends better by imagining how things look from their perspective.	0	1	2	3	4
10. Becoming extremely involved in a good book or movie is somewhat rare for me.	0	1	2	3	4
11. Other people's misfortunes do not usually disturb me a great deal.	0	1	2	3	4

12. After seeing a play or movie, I have felt as though I were one of the characters.	0	1	2	3	4
13. Being in a tense emotional situation scares me.	0	1	2	3	4
14. When I see someone being treated unfairly, I sometimes don't feel very much pity for them.	0	1	2	3	4
15. I believe that there are two sides to every question and try to look at them both.	0	1	2	3	4
16. I would describe myself as a pretty soft-hearted person.	0	1	2	3	4
17. When I watch a good movie, I can very easily put myself in the place of a leading character.	0	1	2	3	4
18. I tend to lose control during emergencies.	0	1	2	3	4
19. When I'm upset at someone, I usually try to "put myself in his shoes" for a while.	0	1	2	3	4
20. When I am reading an interesting story or novel, I imagine how I would feel if the events in the story were happening to me.	0	1	2	3	4
21. When I see someone who badly needs help in an emergency, I go to pieces.	0	1	2	3	4
22. Before criticizing somebody, I try to imagine how I would feel if I were in their place.	0	1	2	3	4

SOC Questionnaire

The following statements are about your feelings in a variety of situations. For each item, indicate how well it describes you by choosing the appropriate number on the scale.

1. I can do just about anything I really set my mind to.

1.	2.	3.	4.	5.	6.	7.
Strongly disagree	Somewhat disagree	A little disagree	Neither agree nor disagree	A little agree	Somewhat agree	Strongly agree

2. Other people determine most of what I can and cannot do.

1.	2.	3.	4.	5.	6.	7.
Strongly disagree	Somewhat disagree	A little disagree	Neither agree nor disagree	A little agree	Somewhat agree	Strongly agree

3. When I really want to do something, I usually find a way to succeed at it.

1.	2.	3.	4.	5.	6.	7.
Strongly disagree	Somewhat disagree	A little disagree	Neither agree nor disagree	A little agree	Somewhat agree	Strongly agree

4. There is little I can do to change many of the important things in my life.

1.	2.	3.	4.	5.	6.	7.
Strongly disagree	Somewhat disagree	A little disagree	Neither agree nor disagree	A little agree	Somewhat agree	Strongly agree

5. Whether or not I am able to get what I want is in my own hands.

1.	2.	3.	4.	5.	6.	7.
Strongly disagree	Somewhat disagree	A little disagree	Neither agree nor disagree	A little agree	Somewhat agree	Strongly agree

6. I often feel helpless in dealing with the problems of life.

1.	2.	3.	4.	5.	6.	7.
Strongly disagree	Somewhat disagree	A little disagree	Neither agree nor disagree	A little agree	Somewhat agree	Strongly agree

7. What happens to me in the future mostly depends on me.

1.	2.	3.	4.	5.	6.	7.
Strongly disagree	Somewhat disagree	A little disagree	Neither agree nor disagree	A little agree	Somewhat agree	Strongly agree

8. What happens in my life is often beyond my control.

1.	2.	3.	4.	5.	6.	7.
Strongly disagree	Somewhat disagree	A little disagree	Neither agree nor disagree	A little agree	Somewhat agree	Strongly agree

9. There are many things that interfere with what I want to do.

1.	2.	3.	4.	5.	6.	7.
Strongly disagree	Somewhat disagree	A little disagree	Neither agree nor disagree	A little agree	Somewhat agree	Strongly agree

10. I have little control over the things that happen to me.

1.	2.	3.	4.	5.	6.	7.
Strongly disagree	Somewhat disagree	A little disagree	Neither agree nor disagree	A little agree	Somewhat agree	Strongly agree

11. There is really no way I can solve the problems I have.

1.	2.	3.	4.	5.	6.	7.
Strongly disagree	Somewhat disagree	A little disagree	Neither agree nor disagree	A little agree	Somewhat agree	Strongly agree

12. I sometimes feel I am being pushed around in my life.

1.	2.	3.	4.	5.	6.	7.
Strongly disagree	Somewhat disagree	A little disagree	Neither agree nor disagree	A little agree	Somewhat agree	Strongly agree

Follow-up assessment 2: additional results.

Table S6. Follow-up assessment 2: sample demographic information.

Statistic	N	Mean	St. Dev.	Min	Max
Age	241	20.689	1.48	18	29
Proportion male	239	0.456	0.49	0	1
Phone Size	230	6.043	0.496	4.7	6.95
iOS	232	0.207	0.406	0	1

Table S7. Follow-up assessment 2: IRI and SOC outcomes.

Item	Components	Neutral video (N=80)	Leadership video (N=81)	Volunteer video (N=80)
IRI (0 – 88)	perspective taking (items 6, 9, 15, 19, 22), empathic concern (items 1, 2, 7, 11, 14, 16), personal distress (items 4, 8, 13, 18, 21), fantasy (items 3, 5, 10, 12, 17, 20)	55.48 (9.44)	55.49 (9.55)	54.63 (10.02)
SOC (12 – 84)	mastery (questions 1 to 4), perceived constraints (questions 5 to 12)	55.71 (9.16)	53.43 (10.26)	54.03 (10.79)

Notes: *p<0.1; **p<0.05; ***p<0.01, based on two-tailed Wilcoxon rank-sum test versus the Neutral video. Mean (SD) values are presented in the table.
